# Application of Metabolomics in Drug Resistant Breast Cancer Research

**DOI:** 10.3390/metabo5010100

**Published:** 2015-02-16

**Authors:** Ayesha N. Shajahan-Haq, Mehar S. Cheema, Robert Clarke

**Affiliations:** Lombardi Comprehensive Cancer Center and Department of Oncology, Georgetown University School of Medicine, 3970 Reservoir Road NW, Washington, DC 20057, USA; E-Mail: bjs.cheema9@gmail.com; clarker@georgetown.edu

**Keywords:** breast cancer, drug resistance, metabolomics, cellular metabolism, cell death

## Abstract

The metabolic profiles of breast cancer cells are different from normal mammary epithelial cells. Breast cancer cells that gain resistance to therapeutic interventions can reprogram their endogenous metabolism in order to adapt and proliferate despite high oxidative stress and hypoxic conditions. Drug resistance in breast cancer, regardless of subgroups, is a major clinical setback. Although recent advances in genomics and proteomics research has given us a glimpse into the heterogeneity that exists even within subgroups, the ability to precisely predict a tumor’s response to therapy remains elusive. Metabolomics as a quantitative, high through put technology offers promise towards devising new strategies to establish predictive, diagnostic and prognostic markers of breast cancer. Along with other “omics” technologies that include genomics, transcriptomics, and proteomics, metabolomics fits into the puzzle of a comprehensive systems biology approach to understand drug resistance in breast cancer. In this review, we highlight the challenges facing successful therapeutic treatment of breast cancer and the innovative approaches that metabolomics offers to better understand drug resistance in cancer.

## 1. Introduction

The fact that cellular metabolism of cancer cells is different from that of normal cells has been known for several decades [1]. “Warburg effect” or increased rate of aerobic glycolysis, rather than the more energy-efficient mitochondrial oxidative phosphorylation, highlights the cancer cell’s needs beyond energy. With aberrantly higher rates of cell proliferation, it is not surprising that rates of aerobic glycolysis, glutaminolysis or fatty acid synthesis are also abnormally higher in cancer cells to keep up with both energy and biomass demands [2,3]. However, our understanding of the complex nature of cellular metabolic pathways in cancer remains incomplete. Changes in gene expression and protein translation can cause robust changes in the cellular metabolite profiles or metabolome. Therefore, comparing the metabolite profile of cancer cells *versus* normal cells can help researchers identify the metabolic changes that promote carcinogenesis. With technological advancement in mass spectrometry, high throughput metabolite profiling (metabolomics) of cancer cells or tumors allows researchers to identify and validate cellular metabolic pathways that contribute to the malignant phenotype. 

Genomics studies investigate differences in sequences in nucleotides that constitute protein coding genes, non-coding DNA and regulatory regions while proteomics studies identify function of proteins in cancer cells compared with cancer cells [4]. Gene expression profiles [5,6,7] and proteomics [8], although expensive, in recent years have provided a glimpse into the complex genetic makeup of breast cancer subtypes and their correlation with survival, chemotherapeutic response or metastatic spread. Metabolomics is the newest layer of “omics” data that is rapidly gaining attention of breast cancer researchers worldwide. The metabolome of a cell comprises of the highly complex biochemical pathways with numerous small molecules or metabolic substrates that include amino acids, sugars, lipids and other bioactive agents. Metabolites serve as chemical byproducts or substrates of naturally occurring biochemical processes and pathways, in a biological system. Metabolomics seeks to quantify the metabolites in the metabolome, and use this data to (in relation to other “omics” fields) eventually diagnose various diseases. Hence the identification of molecular targets that underscore a drug resistant phenotype can be effectively used for developing disease modifying therapeutics. Advances in metabolomics technologies have enabled researchers to design and implement novel strategies in following cancer prognosis and development of customized therapeutics [9,10,11]. Complex signaling associated with cancer phenotypes occurs in the context of interactive networks [12,13] and may be further compounded by drug treatment. Thus, a systems approach using both computational and mathematical modeling may be needed to uncover how the cancer cell responds to external stress and adapts to acquire drug resistance. Precise prognostic tools in personalized medicine are needed not only to identify patients who will benefit from specific treatment options but also to determine dosing strategies to improve drug efficacy. In this review, we discuss the current challenges in drug resistance in breast cancer and what new opportunities metabolomics can provide for researchers.

## 2. Breast Cancer

### 2.1. Breast Cancer Biology and Therapeutic Options

Each year, 1.3 million new cases of breast cancer are diagnosed worldwide, and account for almost 15% of all cancer-related deaths [14]. In the United States, the number of breast cancer cases is projected to increase each year, and therefore, this disease, among other cancers, poses a significant burden to health care and the economy [15]. Breast cancer is a heterogeneous disease [6,16,17] with multiple subtypes and cellular/molecular characteristics, and thus, one of the major challenges for successful treatment in the clinic has been lack of reliable molecular predictors. The standard treatment option for localized breast cancer is surgery or mastectomy with or without radiation while systemic adjuvant therapies (chemotherapy, endocrine therapy or biologic therapy) are used to control tumor growth and improve survival [18]. Various clinical factors including age, menopausal status, lymph node invasion and tumor size are essential in determining the best therapeutic option for a breast cancer patient. Other essential biochemical information required for therapeutic decisions are hormone receptor status including estrogen receptor alpha (ESR1/ER), progesterone receptor (PGR/PR) or growth factor receptor status such as HER2/neu (ERBB2) expression or histological grade determined by immuno-histochemical stains (IHC) [19]. However, minor differences in review of pathology slides can greatly impact clinical decisions and patient care [20]. Moreover, hormone receptor and HER2 status may change with cancer progression and treatment [21,22] necessitating the development of precise biomarkers for breast cancer subtypes that can be monitored in real-time. Gene expression studies carried out over the last two decades studies have recently resulted in the development of gene signatures such as MammaPrint (71-genes) [23] or Oncotype DX^®^ Recurrence Score (21-genes) [24] that can help assign a prognostic score in early breast cancer to determine benefits of adjuvant treatment.

Estrogen and estrogen receptors play significant roles in the development of human breast cancer in 70% of breast cancer cases that are ER-positive [25]. Pharmacological agents and that inhibit estrogen signaling are collectively referred to as endocrine therapy and is commonly used as initial treatment option for breast cancer that is ER /PR-positive. The purpose of such therapy is to block ER activity with antiestrogens such as Tamoxifen or Faslodex/Fulvustrant/ICI [26,27,28,29] or to diminish estrogen-mediated signaling by reducing estrogen synthesis with aromatase inhibitors [30,31,32]. In menopausal women, aromatase inhibitor such as Letrozole/Femara is superior to Tamoxifen as a first-line treatment [33]. HER2-positive breast cancer constitutes about 20% of all types of breast cancer and is characterized by aggressive disease progression and poor prognosis. Targeted therapies for HER2-positive tumors include trastuzumab or pertuzumab, anti-HER2 monoclonal antibodies [34,35], lapatinib, a small-molecule tyrosine kinase inhibitor directed both to HER2 and HER1 [36] or trastuzumab emtansine (T-DM1), an antibody-drug conjugate [37]. Triple-negative breast cancer (TNBC; ER-/PR-/HER2-) accounts for about 15-17% of all types of breast cancer cases but this group has a heterogeneous molecular profile. Antiestrogens and anti-HER2 therapeutics are ineffective in treating TNBC, which remains a subgroup of breast cancer without any specific target. Some TNBCs can be cured by surgery followed by standard chemotherapy (anthracycline/taxane or taxane with carboplatin). Other therapeutic drugs currently in clinical trials for TNBC include agents that are anti-angiogenic, anti-EGFR, poly(ADP-ribose) polymerases (PARP) inhibitors, anti-Src kinase, PI3K and CDKs [38]. 

### 2.2. Drug Resistance in Breast Cancer

Regardless of biochemical subtypes or clinical subgroups, drug resistance in all types of breast cancer remains an unsolved clinical problem. While a vast majority of breast cancers are treated with endocrine therapy, about 40%–50% of these tumors will display de novo or acquired resistance [39,40]. Although various studies using genomics technologies have shed light on the ER-regulated pathways that may contribute to the antiestrogen resistant phenotype [41,42,43], identification of precise biomarkers of antiestrogen responsiveness remains elusive. Proteomic studies have recently uncovered ER-associated co-regulators and transcription factors as possible targets in endocrine resistant breast cancers [8,44]. In HER2-positive breast cancer, treatment with trastuzumab along with standard chemotherapy has significantly improved survival in the past decade [45]; however, resistance remains a critical setback. Increased activity of other HER family members and crosstalk with signaling pathways such as PI3K/AKT are among various other factors contributing to trastuzumab resistance in HER2-positive breast cancer [38]. Chemoresistant TNBC tend to have an aggressive clinical course with early relapse [46]. Master regulators of cellular metabolomics such as MYC, p53 and MTOR are mutated in various subgroups of breast cancer [47,48]. Since MYC, p53 and MTOR are known to impact metabolic pathway, therefore, critical evaluation of cellular metabolic pathways, along with protein function, should be investigated to better understand the pathobiology of breast cancer and drug responsiveness.

While the molecular basis of drug resistance in breast cancer remains unknown, concomitant deregulation of cell death pathways to promote cell survival in response to anti-cancer therapy is evident [13,40,49,50,51]. Research from our group has shown the important role of apoptosis, autophagy and necrosis in cell death in ER+ breast cancer induced by antiestrogen or chemotherapeutic agents [52,53,54,55,56,57,58,59]. Programmed cell death (PCD) pathway such as apoptosis (PCD1), autophagy (PCD2), or necrosis (PCD3) are closely regulated in neoplasia and major molecular inhibitors of these pathways are often overexpressed in tumors. For example, BCL2 [54], NFkB (RELA) [56,57], XBP1 [55], HSP5A/GRP78 [53] MYC [60,61] are known to be overexpressed in breast cancer cells or tumors that are resistant to antiestrogens. In the cell, these molecules not only inhibit cell death, they regulate numerous other processes including cellular metabolism [50]. Moreover, overexpression of these pro-survival molecules allows cancer cells to achieve rapid recovery from disruptions in glucose/ATP levels or amino acid synthesis induced by therapeutic agents. In the normal breast, extensive production of milk proteins is carefully regulated to avoid the endoplasmic reticulum (ER) stress due to an excess load of these proteins and induction of the unfolded protein response (UPR). The UPR protects the cells under normal conditions and helps restore homeostasis. In breast cancer cells, UPR can be triggered by a variety of sources including nutrient deprivation, hypoxia and therapeutic interventions. Both cytotoxic and endocrine therapeutics can induce UPR in different cancer cells [53,62]. Oncoproteins such as MYC that are master regulators of cellular metabolic pathways can also regulate the UPR [63,64]. MYC is increased in antiestrogen resistant cells and tumors [60,61,64]. MYC can increase the dependency of breast cancer cells on glutamine and glucose for cell survival [64]. However, the presence of glutamine in glucose deprived conditions can initiated an UPR-mediated re-programming of metabolic pathways allows cellular adaptation and increased dependency on glutamine that is regulated by MYC and the UPR. The endpoint of UPR induction in cancer cells can be either prodeath or prosurvival and depends on the nature and duration of the stress. Therefore, a complex regulatory mechanism exists to control the UPR in carcinogenesis with key factors from both cell death and survival pathways [50]. 

## 3. Metabolomics as a Promising New Tool in Breast Cancer Research

### 3.1. Current Metabolomics Technologies

Metabolomics is a rapidly emerging field of research that aims to detect and quantify alterations in small-molecule abundance that is known collectively as the metabolome [22,65]. Akin to gene and protein expression that differs across the various cell types of the body, the metabolome is also context-dependent, and varies in organs, tissues and cells, and, importantly, in health and disease processes and external stimuli [66,67]. Metabolomics is the downstream complement of “omics” technologies that span genomics, transcriptomics, RNAseq and proteomics, and thus provides the basis for development of a comprehensive systems biology understanding of stress signaling in injury and disease, which is relevant to many fields such as pharmacology, diabetes, and cancer [68,69]. Traditionally, Nuclear Magnetic Resonance (NMR) was used for metabolomics profiling and early reports of biomarker discovery in breast cancer used this technology [70,71]. However, NMR lacked the sensitivity for detection of low abundance metabolites that could be used as specific and sensitive biomarkers for early detection and staging breast cancer cases. Moreover, in the recent years, there have been dramatic technological advancements in mass spectrometry leading to higher sensitivity and resolution for metabolomic profiling. Many of the problems intrinsic to the NMR-based metabolomics studies thus can be circumvented through the use of liquid chromatography (LC) or gas chromatography in conjunction with high resolution mass spectrometry (MS) [72]. One of the main advantages of mass spectrometry is that constituents of the biological matrix are usually resolved as discreet peaks in a chromatogram that yields an accurate mass and can be subjected to tandem MS that can yield unequivocal identities of large numbers of analytes. These ions representing a unique metabolite are readily amenable to quantification. When combined with ultra-performance liquid chromatography (UPLC), high resolution MS instrumentation can typically yield more than 4000 features that need to be characterized for identification of predictive metabolite markers. The ability to analyze the samples in positive and negative electrospray ionization mode yields complimentary information thus widening the metabolome coverage. Each of these features possesses a characteristic retention time value on the LC column, a mass-to-charge ratio, and an intensity value [73,74]. A typical metabolomic experiment performed using these methodologies can easily generate a large number of data points with high dimensionality. Comprehensive informatics approaches including multivariate data analysis (MDA) methods are then used for data reduction, noise filtration and biomarker selection. One of the main advantages of using GC-MS is the ability to use spectral libraries for unambiguous metabolite identification [75]. However, since the metabolome is highly sensitive to perturbations several aspects have to be carefully considered to minimize variability and low signal to noise that is commonly associated with clinical cohort studies. An important aspect of the study design is to control for pre-analytic variables such as sample collection, storage and the number of freeze thaw cycles that influence the downstream results. 

### 3.2. Metabolites as Powerful Biomarkers of Breast Cancer

Given the high prevalence and mortality associated with breast cancer, there is an urgent need for biomarkers that can be used in the clinic for identifying at risk individuals, for monitoring disease progression as well as for assessing the response to therapy [76,77]. An ideal biomarker should be specific, sensitive be assayable in a cost-effective, high through put manner. Metabolomics has several advantages as a quantitative, high through put technology that can be used to develop predictive, diagnostic and prognostic markers of breast cancer. Early observation by Warburg established that cancer cells switch to a glycolytic phenotype thus reprograming metabolism that promotes cell division and proliferation [1,78]. Subsequently, several studies have shown the role of glycolytic flux in oncogenesis of breast cancer [79,80,81,82]. Given the metabolic reprograming in cancer, it is reasonable to assume that some of these alterations would be stable and amenable to quantitative measurements for diagnostic and prognostic purposes. Several studies have thus used a metabolomic approach to discern alterations in breast cancer that could be potentially used for disease stratification [4,83,84,85,86,87]. Budczies *et al.* found alterations in beta-alanine and glutamine metabolism in estrogen receptor positive (ER+) as compared to ER- breast cancer [88]. Using an NMR approach, Jobard *et al.* reported a panel of metabolites including histidine, acetoacetate, glycerol, pyruvate, glycoproteins (N-acetyl), mannose, glutamate and phenylalanine that could discriminate patients with metastatic breast cancer from those with localized disease with a specificity of 79.8% [89]. Metabolomics has also been used for unraveling diagnostic biomarkers of non-invasive breast cancer which has been comprehensively reviewed [10]. Qiu *et al.* used a quantitative mass spectrometry based plasma metabolomics to identify a lipid panel that could distinguish breast cancer patients from healthy controls [90]. Metabolomics had also been used to study response to neo-adjuvant chemotherapy, tumor microenvironment and response to hypoxia breast cancer [91,92,93,94]. Metabolomics has also helped further the understanding of how central carbon metabolism is altered in tumor tissues as compared to normal [95]. Martinez-Outschoorn *et al.* have reported an association of elevated ketones and lactate with increased “stemness” of breast cancer cells while other metabolomic studies have reported increased oxidative stress in these cells [96]. The term “oncometabolite” is used to describe cellular metabolites that abnormally accumulate in cancer cells and tumors and are associated with malignant phenotype [9,97]. Oncometabolites such as fumarate, succinate, and D-2-hydroxyglutarate can drive oncogenesis partly by regulating epigenetic changes in certain types of cancer [98]. Through metabolomic analysis, Jain et al have identified glycine as an important metabolite that promotes rapid cell proliferation in breast cancer cells [99]. Although a lot of studies in the recent years have successfully utilized a metabolomics approach, future validation studies with independent cohorts would be critical for determining the efficacy and clinical utility of these biomarkers.

### 3.3. Uncovering New Therapeutic Targets through Metabolomics

It is well known that although two individuals may be clinically diagnosed with breast cancer, their tumors’ response to therapy may vary depending on the intrinsic molecular heterogeneity of the tumor [100,101,102]. Thus, understanding the specific biochemical changes accompanying the disease sub type offers an attractive platform for the development of novel therapeutics that can be used to customize response thus improving clinical outcomes in breast cancer [103,104]. The ability to simultaneously measure thousands of metabolites, allows for identification of key metabolic pathways that are under or over represented in breast carcinogenesis. To date, several studies in breast cancer cell models, tumors, serum or urine have been used to understand the underlying causes of breast cancer progression and response to specific anti-cancer therapy (Table 1). These studies support the idea that metabolomics can be a useful tool to differentiate breast cancer subtypes, and also provide a glimpse into the cellular processes in response to anti-cancer therapy. Comparing the changes in metabolites in cancer cells and tumors that are sensitive or resistant to commonly used breast cancer therapies, for example, antiestrogens or taxol, can help investigators determine the biochemical processes that are correlated with cell death or survival. 

Collectively, these studies highlight the importance of metabolomics as a powerful tool to understand the biochemical differences between normal and tumors tissue, and between the various sub-typed of breast cancer. Development of accurate detection tools with metabolites that can serve as biomarkers for disease state or drug responsiveness from serum or urine may provide non-invasive diagnostic approaches in the clinic. These studies also underscore the gaps in the field of breast carcinogenesis and drug responsiveness. While the recent surge in metabolomics-driven research in cancer is commendable, thorough studies involving the contributions of the tumor microenvironment should be included in analysis and validation. Thus, more *in vivo* metabolomics studies for breast cancer subtypes and treatment groups are needed to determine the role of the biochemical pathways that may provide essential insights into the tumor microenvironment and the mechanism of drug resistance leading to recognition of molecular targets that can be used for the development of targeted therapeutics [105].

**Table 1:** Studies involving metabolomics analysis aimed at understanding breast cancer progression and identifying new molecular targets.

Biological materials	Approach	Specific treatment	Metabolic pathways identified	Reference
ER+ and ER- tumor tissues	GC-MS	None	Increase in glutamate, xanthine, beta-alanine in the ER- disease	[88]
MCF7 (ER+)	GC-MS	adriamycin	Increase in glycerol metabolism and decrease in glutathione biosynthesis	[106]
MDA-MB-231 (ER-)	NMR	hypoxia	Increase in glutamate, valine, and leucine and decrease in proline, creatine, alanine	[107]
MCF7 (ER+)	NMR	ascididemin	Increase in citrate, gluconate and polyunsaturated fatty acids and decrease in glycerophospho-choline and -ethanolamine	[108]
serum: early and metastatic breast cancer	NMR	None	Increase in histidine, acetoacetate, glycerol, pyruvate, glycoproteins (N-acetyl), mannose, glutamate and phenylalanine and decrease in alanine	[89]
MCF7 (ER+) and MDA-MB-231 (ER-)	NMR	curcumin +/- docetaxel (dose- and time-response)	Changes in glutathione metabolism, lipid metabolism, and glucose utilization - some biphasic changes depending on exposure	[109]
MCF7 (ER+) and MDA-MB-231 (ER-)	LC-MS	resveratrol	Increased amino acid and arachidonic acid in both cell lines	[110]
serum: recurrent and non-recurrent breast cancer	NMR & GC-MS	None	Changes in amino acids metabolism (glutamic acid, histidine, proline and tyrosine), glycolysis (lactate), phospholipid metabolism (choline) and fatty acid metabolism (nonanedioic acid)	[83]
urine: early-/late-stage breast cancer and normal	NMR	None	Changes in metabolites relating to energy metabolism, amino acids, and gut microbial metabolism	[111]

## 4. Current Challenges in Metabolomics-Based Breast Cancer Research 

### 4.1. Metabolomics Complements Other “Omics” Disciplines in a Systems Biology Approach towards Precision Medicine

A systems biology approach to treatment and research facilitates in lending a holistic view of the intricate relations of various biological systems, in terms of the population or patient in question. Systems biology is a developing research paradigm and has been seen, in some cases, as more effective than the previously more habitual, reductionism [112,113]. It embraces the idea of an all-inclusive perspective that takes into account all biological pathways at work, in coherence with each other and has been reviewed in details [114,115]. It relies on the premise that the pathophysiology of cancer progression results from a malfunction of molecular networks and hence [8,9,10,116,117,118] a comprehensive understanding of pathway based response at different levels of cellular expression, would yield new insights into tumor heterogeneity thus augmenting the personalized medicine paradigm [11,119]. Alterations in specific metabolic pathways can be used not only for understanding the molecular mechanism of disease progression but also for identification of molecular targets that can be used for therapeutic development. Qualitative and quantitative assessment of metabolite levels in urine, blood, needle biopsies or ductal lavage fluids offers promise in identification of predictive biomarkers of breast cancer that can be used for early detection, diagnosis and disease stratification. The challenge of Systems biology lies in effective data integration of metabolomics data with other “omics” data as well as clinical correlates to understand disease progression [120,121]. Initial biomarker discovery studies need to be followed up by large scale validation studies with diverse cohorts, nevertheless metabolomics holds promise for improvising strategies for individualized approach and personalized therapy [122].

**Figure 1:**
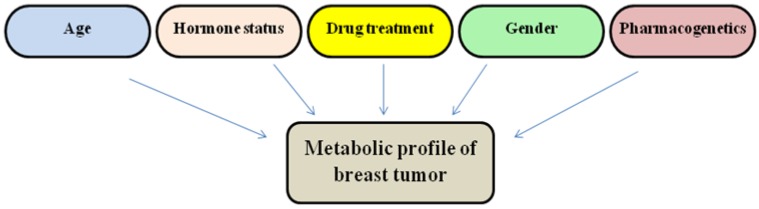
Metabolomics analysis of tumors depends on multiple factors associated with an individual patient.

Metabolomics-based research can be divided into two different categories: non-targeted and targeted metabolomics. Non-targeted metabolomics tries to uncover and identify new chemical compounds that could prove quintessential in understanding the change in a biological systems state of health. These new metabolites, for example could be produced because of a change in the state of the being's existence, environmental factors, *etc.*, and these changes could lead to a diagnoses of the beings condition [123,124]. For example, non-targeted metabolomics of breast tumors from a patient could be a composite of several factors, including age, gender, hormone status, drug treatment and pharmacogenetics (Figure 1). 

### 4.2. Targeting Metabolic Pathways in Cancer

Altered metabolic pathways are rational, potential therapeutic targets. Although the mechanistic details remain unclear, it is becoming increasingly evident that oncoproteins regulate cellular metabolism, such as glycolysis [125] and glutaminolysis [126], to provide energy and substrates to the highly proliferative cancer cell. In addition, increased glycolysis has been linked to drug resistance in cervical cancer cells through pyruvate dehydrogenase kinase (PDK) isoforms PDK1 and PDK3 [127] and through increased lactate production in colon cancer cells [128]. Inhibiting PDK with dichloroacetate (DCA), shifts metabolism from glycolysis to glucose oxidation, inhibits tumor growth and induces apoptosis in several types of cancer [129]. Intermediates generated via glycolysis promote the pentose phosphate pathway (PPP) to generate NADPH and ribose-5-phosphate that are essential for lipid and nucleic acid synthesis, respectively [130]. NADPH is needed to maintain adequate cellular levels of the antioxidant glutathione (GSH), a tripeptide of glutamate, cysteine and glycine that is dependent on glutaminolysis [131]. High levels of GSH have been implicated in chemoresistance in cancer [132]. 

Studies focused on specific enzymes and intermediate metabolites involved in cellular metabolic pathways highlight the importance of these biochemical processes on cell survival and resistance to anticancer therapy. Transketolase (TKT) or transketolase-like protein 1 (TKTL1), enzymes in the PPP, sustains viability of tumor cells [133] and confers resistance to anti-EGFR antibody therapy [134] and imatinib [135]. Hexokinase II (HK2), localized to the outer membrane of mitochondria, is highly expressed in various cancers and can inhibit apoptosis [136] and promote cell proliferation [137]. Glyceraldehyde-3-phosphate dehydrogenase (GAPDH) is overexpressed in cancer [138] and may promote resistance to chemotherapy by inducing Bcl-xL overexpression [139]. Expression of an embryonic M2 isoform of pyruvate kinase (PKM2) promotes tumorigenesis [140] and is regulated by hypoxia-inducible factor-1 (HIF-1) in reprogramming of glucose metabolism in cancer [141]. Specific isoforms of lactate dehydrogenase (LDH) and monocarboxylate transporters (MCTs) are differentially expressed in ER+ and ER- breast cancer cells depending on the cellular demand for glycolysis [142]. Also, the MTOR pathway in cancer cells can detect environmental conditions and adjust cellular metabolic processes by sensing intercellular energy levels through AMPK [143]. Enzymes involved in serine metabolism has helped identify a potential role of serine metabolism in aggressive TNBC [144]. Connecting the expression levels of enzymes widely available gene expression data with metabolites of pathways in different breast cancer subtypes and treatment conditions, therefore, allows investigators to identify critical metabolic pathways that drive a specific phenotype. Thus, knowledge of the metabolic pathways that sustain cancer cell survival within tumors can be used to design better anti-cancer therapeutics to avert drug resistance. 

## 5. Conclusions

Breast cancer is a heterogeneous disease and the recent progress in uncovering the molecular makeup of the disease has guided researchers and clinicians to reject a one-size-fits-all approach to treatment. Successful use of metabolomics in identifying breast cancer biomarkers for specific subtypes or drug responsiveness will provide non-invasive methods to accurately define characteristics of a patient’s cancer in the clinic from body fluids such as blood, urine, sweat or nipple aspirates. Moreover, identification oncometabolites will help target the metabolic pathways that promote cell survival and drug resistance. Efficient management and analysis tools for large volume of data from breast cancer cell models or patient samples and better mode of integration of metabolomics data with transcriptomics and proteomics data to translate the high-throughput information to clinical diagnosis can help accelerate the translation of new findings in the laboratory to the clinic.
